# Smoking among adults in Germany

**DOI:** 10.17886/RKI-GBE-2017-043

**Published:** 2017-06-14

**Authors:** Johannes Zeiher, Benjamin Kuntz, Cornelia Lange

**Affiliations:** Robert Koch Institute, Department for Epidemiology and Health Monitoring, Berlin, Germany

**Keywords:** SMOKING, ADULTS, EDUCATION, HEALTH MONITORING, GERMANY

## Abstract

Smoking poses a considerable health risk and is the leading cause of premature death. Germany has implemented numerous measures (such as tax increases, protection of non-smokers, and cigarette warning labels) to reduce the population’s tobacco consumption. According to the GEDA 2014/15–EHIS survey, 20.8% of women and 27.0% of men aged 18 and over smoke at least occasionally. For both genders, the share of smokers is highest among the younger age groups. Among women and men with higher levels of education, smoking is far less common than among those with lower levels of education. Since 2003, the share of smokers in the adult population has decreased. Compared to other European countries, and in spite of making considerable progress in tobacco prevention policy, Germany still has great potential for improvement in many areas, such as bans on tobacco adverts and tobacco taxation.

## Introduction

In industrialised countries and a growing number of emerging nations, smoking is the single most important preventable health risk and the leading cause of premature death. Globally, tobacco consumption is responsible for around five million deaths annually; including the deaths caused by the effects of passive smoking, this figure rises to nearly six million [1, 2]. Estimates for Germany reckon with around 121,000 smoking-related deaths in 2013, a 13.5% share of all deaths [3]. Smoking contributes to cardiovascular diseases, respiratory diseases, and cancer, among other illnesses [4].

Over the past twenty years, Germany has implemented numerous measures to reduce the population’s consumption of tobacco. Most importantly, between 2002 and 2005, the country significantly increased the tax on tobacco products. Further important steps included legislation to protect people from passive smoking at work, the prohibition of selling tobacco to people under the age of 18, restrictions on tobacco advertising, and federal and state legislation for the protection of non-smokers [5]. Since May 2016, labels on cigarette packs that combine a written warning with what are known as shock pictures have been mandatory in Germany. These must cover 65% of the area on the front and back of cigarette packs. Moreover, laws in Germany regulate the sale and consumption of electronic inhalation products. These measures were accompanied by the national health target for reducing tobacco consumption, an initiative that began in 2003, was evaluated in 2009, and was finally updated in 2015 [6, 7]. Furthermore, in the context of the country’s sustainability strategy, Germany is striving to reduce the share of smokers in the population [8]. At the international level, the WHO’s Framework Convention on Tobacco Control (FCTC) came into effect on 2005 as the first global health agreement, which has since been implemented by most countries, including Germany [3, 9].

**GEDA 2014/2015-EHIS**

**Data holder:** Robert Koch Institute

**Aims:** To provide reliable information about the population’s health status, health-related behaviour and health care in Germany, with the possibility of a European comparison

**Method:** Questionnaires completed on paper or online

**Population:** People aged 18 years and above with permanent residency in Germany

**Sampling:** Registry office sample; randomly selected individuals from 301 communities in Germany were invited to participate

**Participants:** 24,016 people (13,144 women; 10,872 men)

**Response rate:** 26.9%

**Study period:** November 2014 - July 2015

**Data protection:** This study was undertaken in strict accordance with the data protection regulations set out in the German Federal Data Protection Act and was approved by the German Federal Commissioner for Data Protection and Freedom of Information. Participation in the study was voluntary. The participants were fully informed about the study’s aims and content, and about data protection. All participants provided written informed consent.

More information in German is available at www.geda-studie.de

## Indicator

In the GEDA 2014/2015-EHIS questionnaire, the relevant question to measure smoking status is: ‘Do you smoke?’ (answer categories: ‘yes, daily’, ‘yes, occasionally’, ‘no, not any more’, ‘I have never smoked’). Based on these answer categories, the survey subsequently distinguishes between current smokers (daily or occasionally), former smokers, and non-smokers. Previous health surveys determined smoker status in a similar way, which makes it possible to draw conclusions about developments over time and trends [10, 11]. The results are stratified according to gender, age, education, and, for current smokers, according to gender and federal state.

The analyses are based on the data received from 23,960 respondents aged 18 and above (13,108 women, 10,852 men) with valid answers on smoking status. Calculations were carried out using a weighting factor that corrects for deviations within the sample from the German population structure (as of 31 December 2014) with regard to gender, age, district type, and education. The International Standard Classification of Education (ISCED) was used to ensure that the responses provided on educational levels were comparable [12]. A detailed description of the methodology applied in the GEDA 2014/2015-EHIS study can be found in the article German Health Update – New data for Germany and Europe in issue 1/2017 of the Journal of Health Monitoring.

## Results and discussion

**Figure 1:**
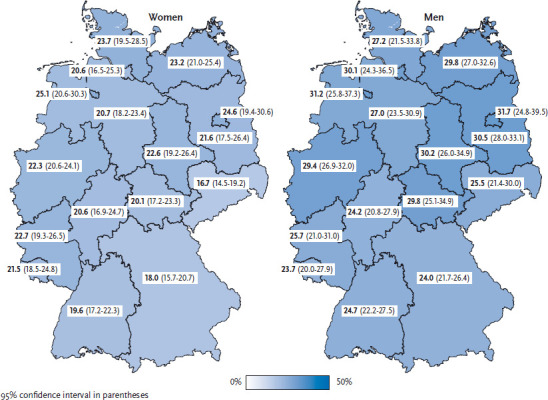
Current smokers according to gender and German federal state (n=13,108 women; n=10,852 men) Source: GEDA 2014/2015-EHIS

Currently, 20.8% of women and 27.0% of men in Germany smoke at least occasionally (Table 1 and Table 2). 52.6% of women and 38.0% of men have never smoked. Among both genders, the share of current smokers is highest in the younger age groups. The percentage of male smokers begins to drop at the age of 45. A significant drop in the percentage of female smokers does not occur until age 65. Among both genders, smoking is far less widespread in the groups with higher levels of education than those with lower levels. With the exception of the 65-plus age group, where no significant differences regarding the educational level appear, this clear link between smoking and education is evident across all other age groups. Moreover regional differences exist in the percentage of smokers in the population. The percentage of male smokers is highest in Saxony-Anhalt and lowest in Bavaria. The percentage of female smokers is lowest in Saxony and highest in Bremen. The percentage of smokers tends to be higher in the north than in the south, higher in the east than in the west, and higher in the federal city-states than in the territorial federal states (Figure 1). In EU member state comparison, Germany is in the middle third for female smoking prevalence and the lower third for male smoking prevalence. A more detailed description of the German results in European perspective is included in the article Health-related be haviour in Europe - a comparison of selected indicators for Germany and the European Union in this issue of the Journal of Health Monitoring [13]. Data from previous Robert Koch Institute (RKI) health surveys reveals that, among the adult population, the percentage of female smokers has dropped by a good eight percentage points and of male smokers by a good eleven percentage points since 2003 [14]. Other surveys, such as the micro census and the Epidemiological Survey of Substance Abuse (ESA), also indicate a decline in the number of adult smokers [15, 16]. Most noteworthy is that ever fewer adolescents are taking up smoking. According to data from the Federal Centre for Health Education, the percentage of 12- to 17-year-old girls who smoke at least occasionally declined from 23% to 8% between 2004 and 2015, and among boys in the same age group from 24% to 8% [17]. Results from the RKI’s German Health Interview and Examination Survey for Children and Adolescents (KiGGS) [18, 19] and the international Health Behaviour in School-aged Children (HBSC) survey [20] equally indicate a clear decline in the prevalence of smoking among adolescents. The impact of the increased use of electronic inhalation products (e-cigarettes) and the entry of the large tobacco corporations into this market remains to be seen. For those engaged in tobacco prevention, the damaging or beneficial effects regarding smoking cessation of this group of products remains a highly controversial issue [21].

In spite of Germany’s progress in tobacco prevention policy, much room remains for further progress in numerous fields. Out of the 35 countries assessed in the tobacco control scale, which compares countries with regard to their efforts in tobacco prevention policy, Germany currently ranks second to last [22]. Germany therefore has great potential for improvement, in particular concerning taxation, smoke-free areas, bans on advertising, prevention campaigns, and providing people with support to quit tobacco.

## Key statements

- 21% of women and 27% of men aged 18 and over smoke at least occasionally.
- Since 2003, the share of smokers in the German population has decreased.
- Among both genders, the share of current smokers is highest in the younger age groups.
- Among both women and men, smoking is much more widespread in groups with lower levels of education.

## Figures and Tables

**Table 1:** Smoker status among women according to age and educational status (n=13,108) Source: GEDA 2014/2015-EHIS

Women	Smokers (daily or occasionally)		Former smokers		Non-smokers	
	%	(95% CI)	%	(95% CI)	%	(95% CI)
**Women total**	**20.8**	**(19.9-21.7)**	**26.6**	**(25.6-27.6)**	**52.6**	**(51.4-53.8)**
**18-29 Years**	28.4	(26.3-30.7)	15.9	(13.9-18.0)	55.7	(53.0-58.4)
Low education	34.7	(28.6-41.4)	16.7	(12.2-22.6)	48.5	(42.2-54.9)
Medium education	28.3	(25.5-31.2)	15.7	(13.4-18.3)	56.1	(52.8-59.2)
High education	19.5	(15.8-23.8)	15.6	(12.2-19.8)	64.9	(59.6-69.8)
**30-44 Years**	26.9	(24.8-29.1)	27.0	(24.9-29.1)	46.1	(43.9-48.4)
Low education	37.0	(30.5-44.0)	24.0	(18.0-31.2)	39.0	(32.4-46.1)
Medium education	29.8	(27.0-32.8)	27.1	(24.6-29.8)	43.0	(40.2-46.0)
High education	14.2	(11.9-17.0)	28.5	(25.1-32.1)	57.3	(53.2-61.3)
**45-64 Years**	24.2	(22.8-25.6)	32.4	(30.8-34.0)	43.4	(41.6-45.2)
Low education	29.8	(25.8-34.1)	30.6	(26.8-34.6)	39.7	(35.4-44.0)
Medium education	25.3	(23.5-27.2)	32.8	(30.8-34.9)	41.8	(39.6-44.1)
High education	15.1	(13.3-17.2)	32.7	(30.3-35.3)	52.1	(49.4-54.8)
**≥ 65 Years**	6.8	(5.8-7.9)	24.9	(22.9-27.0)	68.3	(65.9-70.6)
Low education	5.9	(4.6-7.5)	20.1	(17.4-23.0)	74.0	(70.9-76.9)
Medium education	7.5	(6.1-9.2)	28.3	(25.2-31.5)	64.2	(60.7-67.7)
High education	5.9	(4.0-8.5)	29.4	(24.7-34.6)	64.7	(59.3-69.8)
**Total (women and men)**	**23.8**	**(23.1-24.6)**	**30.7**	**(29.9-31.5)**	**45.5**	**(44.6-46.4)**

CI=confidence interval

**Table 2:** Smoker status among men according to age and educational status (n=10,852) Source: GEDA 2014/2015-EHIS

Men	Smokers (daily or occasionally)		Former smokers		Non-smokers	
	%	(95% CI)	%	(95% CI)	%	(95% CI)
**Men total**	**27.0**	**(25.9-28.1)**	**35.0**	**(34.0-36.1)**	**38.0**	**(36.9-39.1)**
**18-29 Years**	35.1	(32.1-38.3)	12.3	(10.4-14.5)	52.6	(49.4-55.7)
Low education	40.6	(33.7-47.9)	14.8	(10.6-20.2)	44.6	(37.8-51.6)
Medium education	34.5	(30.8-38.4)	11.8	(9.6-14.5)	53.7	(50.0-57.3)
High education	27.4	(22.0-33.6)	9.9	(7.0-13.8)	62.7	(56.2-68.7)
**30-44 Years**	35.7	(33.2-38.3)	28.3	(25.9-30.8)	36.0	(33.5-38.6)
Low education	48.1	(40.4-56.0)	23.1	(16.9-30.8)	28.7	(21.9-36.7)
Medium education	37.6	(34.2-41.1)	31.4	(28.1-35.0)	31.0	(27.7-34.5)
High education	26.4	(23.2-30.0)	24.8	(21.6-28.4)	48.7	(44.9-52.5)
**45-64 Years**	28.3	(26.7-30.1)	37.8	(36.1-39.6)	33.8	(32.2-35.6)
Low education	37.5	(32.6-42.7)	36.5	(31.0-42.4)	26.0	(21.5-31.0)
Medium education	31.5	(29.1-34.0)	38.6	(36.0-41.3)	29.9	(27.5-32.5)
High education	19.4	(17.4-21.6)	36.4	(33.8-39.2)	44.2	(41.4-46.9)
**≥ 65 Years**	9.2	(8.0-10.4)	55.5	(53.2-57.7)	35.4	(33.3-37.5)
Low education	8.6	(5.9-12.2)	54.2	(48.6-59.7)	37.2	(32.1-42.6)
Medium education	9.3	(7.6-11.3)	56.5	(53.2-59.7)	34.2	(31.3-37.3)
High education	9.3	(7.6-11.3)	53.8	(50.4-57.1)	36.9	(33.9-40.0)
**Total (women and men)**	**23.8**	**(23.1-24.6)**	**30.7**	**(29.9-31.5)**	**45.5**	**(44.6-46.4)**

CI=confidence interval
